# Long COVID Patient Symptoms and its Evaluation and Management

**DOI:** 10.31729/jnma.6355

**Published:** 2021-08-31

**Authors:** Deepak Sundar Shrestha, Richard R. Love

**Affiliations:** 1 Department of Internal Medicine, People's Dental College and Hospital, Kathmandu, Nepal; 2 Department of Computer Science, Marquette University, Milwaukee, Wisconsin, United States of America

**Keywords:** *chronic fatigue syndrome*, *COVID-19*, *hypothalamic hormones*, *immune system diseases*

## Abstract

While the acute case burdens and deaths from the COVID-19 pandemic (in Nepal approaching 700,000 and 10,000 respectively) have been costly, the characteristics and potentially huge dimensions of the chronic disease sequelae of this infectious disease are only slowly becoming apparent. We reviewed Pub Med, major medical meeting and medical journal, and investigative journalist materials seeking to frame and describe COVID-19 chronic disease. The consequences of COVID-19 infections follow major organ damage, and induction of immunological and hormonal systems dysfunction. The first injuries are consequent to direct viral effects on tissues, and vasculitis, endothelialitis, thrombosis and inflammatory events. Pulmonary, cardiac, brain, and kidney tissues incur function-limiting damage, with dyspnea, arrythmias, decreased exercise capacity, cognitive dysfunction, and decreased glomerular filtration rates. The second process is characterized by immune dysregulation and autoimmunity, and dysfunction of hormonal regulation systems, with high, fluctuating levels of physical and mental fatigue, multiple-site pain and ache, and non-restorative sleep, in 10-30% of cases. This communication proposes evaluation and management of chronic COVID-19 patients with efficient assessment of commonest symptoms, targeted physical examination and organ function testing, and interventions based on specific organ functional status, and experience with similar chronic immune syndromes, such as myalgic encephalomyelitis.

## INTRODUCTION

Worldwide, patients have called attention first, before health organizations and professionals, to the months-later persistence and development of symptoms and signs of serious illness following infection with COVID-19.^1,2^ The term "long COVID" was first used in Italy by patients, and subsequently acknowledged by the World Health Organization in September, 2020.^3^ Physicians have described the illnesses as 'Post-acute COVID".

## LITERATURE REVIEW

Seeking to frame and describe COVID-19 chronic disease as bases for patient clinical evaluation and management, we reviewed Pub Med, major medical meeting, major medical journal, and investigative journalist materials.

## FINDINGS

### Acute biology

The COVID-19 coronavirus spike protein facilitates entry of the virus into tissue cells engaging angiotensin-converting enzyme 2 (ACE2) as an entry receptor.^45^ Because of the presence of ACE2 receptors throughout body tissues with almost universal organotropism, direct and indirect COVID viral effects occur in multiple organ systems, of particular note the hematological and coagulation systems.^46^ At this time, the delayed pathophysiological disturbances are incompletely characterized, with investigators offering multiple models.^3^ For long COVID patients then, their illnesses pictures are consequent to two broad pathophysiologic sequences and timelines: acute organ injury, and later onset chronic immune and hormonal homeostatic regulatory dysfunction. This communication will focus on the later sequelae, because of apparent greater prevalence in populations.

### Characteristics of acute and post-acute COVID infection

Critically, the lungs, heart, brain, kidney, and hematologic organ systems are acutely impacted by infective direct and indirect effects through mechanisms producing vasculitis, endothelialitis (from viral binding to ACE2 receptor), thrombosis from cytokines (storm) and chemokines, inflammatory markers-D-dimer and fibrinogen and Mast cells.^4-11^ These consequences produce the symptoms and signs with acute and potentially permanent, organ damage, that of necessity have been the focus of clinical care.^12-18^ The available details of specific major organ disease-associated symptoms over time are critical to thoughtful evaluation and management of post-COVID patients (Table 1).

**Table 1:** Major body organ systems affected by acute COVID-19 infection, specific tissue effects, and their functional consequences.^4,6^

Organ system and effects	Functional consequences
Pulmonary	
Macro and micro-vascular thromboses	Dyspnea
Cardiac	
Direct myocardial cell injury	Cardiac dysfunction with biventricular myopathy
Endothelialitis	Decreased physical exertion capacity Arrhythmias
CNS	
Stroke	Cognitive and motor neurological deficits
Encephalopathy	
Neurodegenerative effects	Increase in mood disorders
Renal	
Proteinuria	Chronic kidney disease with decreased glomerular filtration rate
Electrolyte abnormalities	
Hematologic	
Coagulation disturbances with arterial and venous thromboses	Myocardial infarction Stroke

Complex multifactorial syndrome(s) expressed over months with immune dysfunction and autoimmunity, hormonal and renin-angiotensin-aldosterone system disturbances

Beginning months into the COVID pandemic, first through investigative journalist articles, some patients reported the development of new symptoms weeks to months after apparent recovery from the acute illness.^2^ Perhaps earlier, it was very evident that COVID-19 illness was not at all like seasonal influenza, where over 90% of individuals recover completely within 14 days. The recovery times for the majority of COVID patients extend to six to nine weeks. Also, at about the same time into the pandemic—mid-year, 2020—first published reports about recovery patterns noted high frequencies of systemic symptoms.^19^

### Post-acute COVID-19 symptoms

In a sentinel report in 143 hospitalized patients 60 days after diagnosis, dyspnea was present in 43%. The other symptom data in this report were perhaps even more telling: only 13% of patients were symptom-free, and fatigue, joint and chest pain were reported by 53%, 26% and 22% of patients respectively.^19^ Among 279 hospitalized patients 3 months later, 55% reported fatigue and 31% sleep disorder.^20^ Two further study reports add disturbing features to this systemic chronic illness picture. In 201 patients, only 18% of whom had been hospitalized, 140 days after diagnosis 99% had 4 or more symptoms; 42% had 10 symptoms; fatigue, muscle aches and headache were common.^21^ Finally, in large Chinese report, at 6 months following hospitalization, 76% of patients had at least one symptom, 63% fatigue or muscle weakness (significantly in excess of apparent pulmonary or cardiac organ signs), 26% sleep difficulties, and 23% anxiety or depression.^22^ Thus, these reports suggest high levels of new and sustained symptoms appearing independent of acute illness severity, several months after diagnosis. Additionally, anecdotally, patients report late developing cognitive issues, acutely developing dementia and dysautonomia, with autonomic nervous system symptoms: increased heart rate, temperature sensitivity and sweating, dizziness and lightheadedness on standing suddenly.^3^ Lay data support the emerging picture that nonhospitalized patients do develop long-term symptoms, and in particular, that young-middle-aged women may be more affected.^1-3,23^ In general, however, at this time, the majority of the scientifically peer-reviewed data about long-term symptoms, concern patients who have been hospitalized, and thus patients who were severely ill in the acute disease phases.

### Patient laboratory data in post-acute COVID patients

Specific laboratory test observations seem relevant to the emerging picture of long-term symptoms post COVID-19 infection. First, lymphopenia characterizes the acute infection, suggesting a significant immune system impact.^4,6^ Further, preliminarily, high levels of autoantibodies and a strong T cell response, particularly in women, have been found.^3,23^

Data regarding long-term non-COVID post-infection symptoms and syndromes

In a prospective cohort study of 253 patients with different viral infections followed for 12 months: "Prolonged illness characterized by disabling fatigue, musculoskeletal pain, neurocognitive difficulties, and mood disturbance was evident in 29 (12%) of 253 participants at six months, of whom 28 (11%) met the diagnostic criteria for chronic fatigue syndrome".^24^ Ten percent of Lyme disease patients (spirochete bacterial-caused) develop persistent symptoms—fatigue, pain, and dysautonomia, in women more than men.^25^ These frequencies of persistent symptoms are also seen following laboratory confirmed infectious mononucleosis, an Epstein-Barr viral condition. In a prospective series of 142 patients with infectious mononucleosis, 12% (n=17) reported continuing illness characterized by fatigue and poor functional status at 6 months; female gender was the strongest predictor of absence of recovery.^26^ In a second prospective cohort study of 250 patients with mononucleosis, in the most conservative interpretation, nine percentage of patients had "Chronic Fatigue Syndrome'" (CFS) at six months; in follow-up detailed analyses by various CFS definitions, the range was 16-19%.^27,28^ Patients with other triggering infections: viral hepatitis and Q fever are cited as developing CFS at similar doubledigit percentages.^29,30^ In a series of 369 SARS-1 patients, chronic fatigue was reported in 40% (27% met criteria for a diagnosis of Chronic Fatigue Syndrome) and 40% had active psychiatric illness, three and half years after diagnosis.^31^ A case-control study of 22 SARS-1 patients one to three years after diagnosis reported fatigue, muscle pain, depression, and sleep disturbance in "a syndrome which overlaps with Chronic Fatigue Syndrome" (CFS).^32^ A third SARS-1 study of hospitalized patients is remarkable for the 20-30% of cases who were unable to work 2 years later.^33^ A meta-analysis confirms these findings for SARS-1 and extends them to Middle East Respiratory Syndrome (MERS).^34^ A possible confounding issue is that anecdotally patients have reported developing CFS-like syndromes after treatment with fluoroquinolone antibiotics.

These multiple foregoing reports are in the context of a long-known, but neglected syndrome called chronic fatigue syndrome (CFS) or myalgic encephalomyelitis, characterized and diagnosed significantly more frequently in women, solely on the basis of a constellation of fluctuating symptoms, majorly fatigue, multi-site aches and pains--notably joint and head, non-restorative sleep, malaise with associated loss of motivation, cognitive function deficits, depression (considered a brain inflammation symptom) and shortness of breath on minimal exertion, of uncertain etiology, but thought to be post-viral or bacterial infections.^35^ There has been very limited evidence of specific genetic signatures associated with CFS.^36^

### Biological models for post infectious and post-acute COVID syndromes

The complexity of suggested models for postinfectious CFS syndrome is daunting. Viral triggers (particularly Human Herpsevirus-6 and -7) persistent inflammation, poor antiviral responses, and evidence of chronic viral reactivation, with disrupted immune surveillance, all have been suggested with consequent neuroinflammation (possibly of autoimmune sources). Oxidative stress in the central nervous system, as well as a complex homeostatic imbalance of autonomic, endocrine hypothalamic-pituitary and renin-angiotensin-aldosterone, and metabolic-cell danger response and mitochondrial systems have been also posited, particularly as sustaining the immune system induced dysfunction.^35,37-40^

### Summary of the post-acute COVID clinical illness picture

An iceberg analogy is appropriate in describing the emerging picture of post-COVID illness: what we are seeing and identifying in post-COVID patients is very likely but a fraction of what acute organ injuries occur, many of which can lead to both long-term organ-compromise, and long-term complex immune and homeostatic dysfunction persistent infectionlike syndrome(s) with profoundly disabling symptoms and impaired functional levels. The implications for management and interventions are that we need to specifically determine which consequences are occurring in individual patients. It is striking that apparently men are more susceptible to the COVID infection (this is certainly true in Nepal), but in western countries more women are presenting with long-COVID. In high-income country populations, at six months, disability for work, physical and/or cognitive percentages figures range from 10-30%. The higher percentages are suggested by SARS-1, organ failure problems post COVID, and some rigorous post-COVID patient data.^22,31,32^ The at-least 10% figure must be considered firm, given such figures from the spectrum of other post infectious data, but limited data indicate considerable heterogeneity of long-COVID symptoms across countries.^24-29^ Incomplete as the COVID-specific long-term effects data may be, regional differences should be expected because the acute case incidence, mortality and all-cause mortality rates are so dramatically different among countries. These rates in South Asian countries appear to be but one tenth of those in western countries. Mutant variants of COVID, and multiple co-variates may impact expression of long-term symptoms and syndromes. For examples: 1. Altitude effects; 2. Nutrition—Ayurvedic nutrition with dietary immune and antiviral constituents: such as turmeric, cumin, fenugreek, ginger, garlic, and monolaurin. 3. Multiplier effects from air pollution may all have general health impacts.^41^ 4. BCG vaccination widely provided in Nepal, may be immune-protective.^42,43^

## DISCUSSION

### Post-acute COVID individual patient evaluation

**Symptom screening**

It is best to focus asking following questions; by rating how severe the symptoms have been over the last three days: Worst pain/ache, pain/ache according to location (Muscles, Back/whole body, Head, Joints, Chest), mental and physical fatigue/tiredness, poor unrefreshing sleep, fever and/or chills, shortness of breath, cough, feeling sad/depressed, anxious/worried, rapid or irregular heartbeat, change in sense of smell, change in sense of taste, mental confusion or disorientation, difficulty thinking and concentrating, difficulty remembering, difficulty in word-finding, lack of motivation, numbness in fingers or toes, light-headedness or dizziness on standing, heat or cold intolerance, reduced physical activity, increased sensitivity to sound or light, increased fatigue the day after more-than-usual physical or social activity and any other ongoing symptoms the patient associates with COVID infection.

Notable features of these questions are that, items are based on both organ damage and long-term complex biology-created symptoms; translation-wording considerations have governed item language choices-for example combined word questions; the fluctuations of symptoms have led to seeking three-day assessments; patient preferences for limited choices have led to a six-point severity scale; and the instrument brevity—completable in five minutes—may increase response rates and their quality. In use with over 2500 Nepali men and women, all of the instrument questions (in English and Nepali versions) have been understood and readily answered by almost all individuals interviewed. Some broad and preliminary impressions from our data are that one quarter of Nepalis have not recovered from acute COVID at 3 months, but that significant lessening of symptoms occurs between this time and 6 months, such as that this later time at least 10% are still significantly symptomatic. Fatigue and pain/ache are the most common persistent symptoms, but practically all of the symptoms inquired about have been more common in COVID-infected patients than in those not reported to have suffered this illness.

### Focused symptom screening and history

Use of this questionnaire, together with a review of major patient medical problems and diagnoses, habits (especially tobacco), and medications (particularly statins, ACE inhibitors and diabetic drugs), can efficiently lead to consideration of appropriate additional data.^4^ Specifically, reported shortness of breath suggests use of the modified Medical Research Council (mMRC) dyspnea scale, and oximetry.^44^ Reduced physical activity may be evaluated with the six-minute walk test (6-MWT) for aerobic capacity and endurance; this evaluation is more appropriate in the absence of chronic fatigue syndrome-like symptoms, when cardio-pulmonary dominant organ dysfunction seems present.^45^

Cognitive function screening tests are suggested if screening questionnaire cognitive function items get scores of three or more; with tools such as the Mini-mental state exam (MMSE).^46^ Screening tests for anxiety and depression are appropriate if scores for these questions with screening are three or more. If severity scores for fatigue, worst pain or aches, poor sleep, lack of motivation, reduced physical activity, and cognitive functions of three or more on a six point Likert scale are reported, serious consideration should be given to getting a picture of patient's functional status for activities of daily living, using questions such as in a usual day, how many hours is spend on sleeping at night, sleeping in the day, resting-non-sleeping in the day, in social interaction in the day and in activities in a chair in the day. It is worth exploring if health problems interfere with the patient doing the usual work as before having COVID and if there are difficulties in simple tasks such as walking upstairs, eating, doing household tasks, going out of the house for activities, and taking care of children. Also, difficulties in taking care of oneself and need for assistance with home tasks should be explored.

These questions are specifically selected from among 90 items of the Common Data Elements for the US NIH suggested for chronic fatigue syndrome patient evaluation.^47^

### Targeted Physical examination

Again, symptom screening questionnaire answers should clearly suggest targeted organ system examinations for pulmonary, cardiac, and neurological and psychiatric signs.

Targeted organ system laboratory and imaging testing based on symptoms, vital signs, and functional testing

There are obviously many laboratories or imaging studies that may be appropriate in individual post COVID patients. Here we choose to highlight particular studies.

Assessment for COVID reinfection and antibody response. The durations and magnitudes of antibody responses following acute COVID and the implications of any specific data and interpretation of antibody response levels is poorly defined. In patients with indicators of incomplete immune response to COVID infection (persistent fever, continuing pulmonary infectious symptoms and signs, apparent secondary infections) specific anti-viral treatment with agents such as Remdesivir should be considered.^16,17^

### Assessment for persistent and secondary infections.

Consider assessment of C-reactive protein (CRP) a general measure of inflammation and Glycoprotein acetylation (GlycA) a marker of systemic inflammation.^48^

The use of smell cards for evaluation of reported loss of smell.

Thyroid function tests to rule out hypo- or hyperthyroidism.

A complete blood count to evaluate for anemia in particular.

Detailed pulmonary system evaluation with spirometry, Diffusing Capacity of the lung for Carbon Monoxide (DLCO,) and chest X ray.

Cardiac evaluation with electrocardiography (EKG) rhythm monitoring and QT interval assessment; Echocardiography (ECHO) for wall motion abnormalities, pericardial effusion and right and left ventricular dysfunction.

Hemoglobin Alc assessment because of metabolic syndrome development.

Renal function assessment with blood urea nitrogen (BUN), creatinine, estimated glomerular filtration rate (GFR)

Joint imaging to evaluate for inflammatory component, possibly with ultrasound.

It may become critical over time to develop efficient, patient-centered telemedicine evaluation protocols.

### Patient management

**General issues**

The long COVID syndromes and symptoms are real, but frequency, modifiers, and specific symptom complexes in Nepalis are undefined, and subject to current research. When possible, patients and families should be encouraged to explore on-line educational resources about chronic fatigue syndrome (https://mecfscliniciancoalition.org).

With clearly identified multi-organ system symptoms of significant severities, a team of specialists is needed if at all possible. This assessment has led to the development of Post-COVID Clinics/Centers. Based on the development of data about the frequency of long COVID in Nepal, the creation of limited numbers of such centers with tele-medicine capacities should be explored.

For symptom-defined CFS, most critically, rest and pacing of activities are essential. Exercise is not helpful and low levels of activity, which exceed a patient's physical and mental exertion thresholds may lead to drastic worsening of symptoms.^33^

Immunization status review and immunization for COVID are worthwhile and indicated to prevent reinfection, and possibly to boost immune system management. Anecdotally, some patients with CFS or poor COVID recovery report remarkable improvement following immunization, which may because of direct effect of the mRNA in vaccines of those types or because of induced interferon production. It has been observed that women develop stronger immune responses to vaccines than men, with increased antibodies and T cell responses.

Immunizations against secondary infections for pneumococcus, influenza and haemophilus. Theoretically there are risks of over-stressing patients' already dysfunctional immune systems with such immunizations, and anecdotally some CFS patients have suffered precipitous exacerbation of symptoms following immunizations. In individual patients, particularly those with dominant pulmonary organ symptoms and signs of deficits, these risks may be exceeded by the potential benefits.

In patients with a history of or documented poor antiviral function, immunomodulatory treatment with Inosine Acedoben Dimepranol (Imunovir).

Smell and taste sensory disturbances are very common and may be limited by use of supplemental zinc.

Patients with autonomic system disturbances, particularly postural hypotension, may benefit from daily oral electrolyte-balanced fluid replacement. In patients with severe hyperadrenergic states, consideration of low dose beta blocking drugs or fludrocortisone is appropriate.

Specific interventions by major organ associated clusters of symptoms

Pulmonary (dyspnea, cough, objective evidence of physiological deficits by mMRC grade, oximetry, spirometry, DLCO, chest Xray)-targeted treatments. Disturbance of breathing mechanics has been suggested to occur, and breathing exercises may help this problem, as with yoga.

Cardiac (Irregular or rapid heartbeat; reduced physical activity level presumed on cardiac basis; objective evidence of physiological deficits by EKG, ECHO): targeted treatments.

Neuroinflammatory, with immune dysfunction and autoimmune activity (mental and physical fatigue, poor unrefreshing sleep, anxiety, lack of motivation, diffuse aches and pains including headache, depression, cognitive deficit symptoms, increased sensitivity to sound or light, increased fatigue the day after more-than-usual physical or social activity, detailed reports of poor functional status in carrying out activities of daily living.

With severe anxiety, depression, or cognitive function deficits, consider first confirmatory neurological/psychiatric dysfunction data from mini-mental state exam (MMSE), or other screening tools, and then specialist consultation.

With constellation of these symptoms consistent with chronic fatigue syndrome, based on severity consider:

Anti-inflammatory supplements: Coenzyme Q-10, 100200mg/daily; N-acetylcysteine 1200 mg/day; Fish oil omega 3 fatty acids 600 mg daily; Acetyl-L-Carnitine 500 mg. daily; Alpha-Lipoic acid 400 mg daily.^49,50^ Nanocurcumin.

More specific neuro-inflammatory treatments:

*Naltrexone for better sleep and widespread aches; start with a very low dose 0.1-0.5mg in the evening. ^51^

*Glutathione, most effectively as a nasal spray

*Luteolin 100mg p.o. q.d. ^52^

*Minocycline;^53^

Other anti-inflammatory drugs with various, but limited levels of data on efficacy in this condition: Aspirin and other NSAIDs—systemic and topical, colchicine; Pregabalin, Duloxetine, Gabapentin.

Herbal and indigenous, Ayurvedic remedies: Boswelia, turmeric.^54,55^

Serotonin-norepinephrine reuptake inhibitors (SSRIs): these drugs, particularly for example Fluvoxamine, are often recommended, but very definitely require close monitoring because of a spectrum of very serious adverse reactions.

Vitamin C has been suggested to be a specific intervention for fatigue. ^56^

Finally, as a general treatment for long-COVID with major CFS symptomatology, some authors have suggested Ivermectin. The available evidence for benefits from this intervention is limited and controversial and a recent rigorous study has suggested marginal, if any, benefits. ^57^

Dominant auto-immune (Pain and aches—often primarily in muscles and joints, fever, fatigue, temperature sensitivity, fast heartbeat, numbness and tingling in fingers and toes, muscle weakness, and non-specific gastrointestinal symptoms), Consider: Rituximab and cyclophosphamide.

Mast cell activation-like symptoms. For patients with allergic and anaphylactic symptoms—hives, angioedema, and wheezing—non-sedating antihistamines and mast cell-stabilizing agents like ketotifen are appropriate.

## WAY FORWARD

Richard Horton, the editor of The Lancet has written that for much of the natural history of the COVID pandemic, worldwide leaders and doctors have: underestimated the avalanches of population impacts, ignored evidence of the dangers of such a pandemic, communicating poorly and un-transparently, and made too many assumptions.^58^ He is writing about the acute disease phase of this pandemic, but the data presented here clearly indicate that we will and are experiencing a known multidimensional and multi-system disturbing chronic phase, with which we must try to avoid the same mistakes. We may not have second chances. We must not "gaslight"—that is down-play—the frequent serious physical symptoms, and long-term consequences of COVID infections and attribute of these reported symptoms to anxiety or panic attacks or conversion disorder, for trust is the cardinal requirement of scientific, patient-centered and ethical medical care.^59^ We have to respond in agile and creative ways to these chronic phase challenges and all of their consequences. As Horton has emphasized, one huge and major lesson from the acute phase experiences in different countries has been that consistent messaging is critical in achieving the best outcomes for populations. To that end, regarding long post-acute COVID, we suggest the listed public health messages (Table 2). Nationally and locally relevant news items and available multimedia delivery are key to developing trust.

**Table 2:** Public health messages about recovery from COVID-19 and "Long COVID".

With the acute illness phase of COVID, most patients recover within 6 weeks, but some patients' recovery may take months.
Some patients, particularly those who were very ill, may suffer lung, heart, and brain injuries with ongoing shortness of breath, reduced exercise capacity, fast or irregular heart­beat, problems thinking and remembering, and anxiety and depression. Together, these serious symptoms have been called "Long-COVID". Doctors can help control these problems.
Some other patients—how many we don't yet know—get better after COVID, and then weeks to months later develop new symptoms of fatigue and increased fatigue a day after physical or social over-exertion, non-restorative sleep, lack of motivation, headache, joint pains, depression, and new shortness of breath. These new symptoms, are also called "Long COVID", are real.
Advocacy by families and health professionals for recognition and care for long COVID is needed.
